# Seventh Annual Meeting of the Military Tract Medical Association

**Published:** 1872-06-15

**Authors:** Herbert Judd

**Affiliations:** Sec’y.


### Society Reports.

### SEVENTH ANNUAL MEETING OF THE MILITARY TRACT MEDICAL ASSOCIATION.

Pursuant to adjournment the Seventh Annual meeting of the Military Tract Medical Association convened in Masonic Hall at 10 o'clock. In the absence of the President, Dr. E. L. Phillips, of Galesburg, was called to the Chair. Drs. Reese, McClelland and Klingburg, as board of censors, reported the names of Drs. Bunce, Heller, Sapp, and D. T. Brown for membership. The association received them by unanimous vote. The regular order of business was left until the afternoon session.

Dr. W. L. Cuthbert then offered a series of resolutions concerning abortion, which, after some discussion, were referred to a committee consisting of Drs. Cuthbert, Reese, Hurd, Bacon and Bunce. Upon the retirement of the committee the association adjourned until two o'clock.

AFTERNOON.—The association re-assembled with Dr. Phillips in the Chair. On motion, the association proceeded to the election of officers, with the following result: Dr. John W. Hensley, of Gates City, President; Dr. M. A. McClelland, of Knoxville, Vice President; Dr. Herbert Judd, of Galesburg, Secretary and Treasurer. Dr. Hensley returned thanks on taking the Chair in a brief address, promising to discharge the duties to the best of his ability. He then called for the reports of the committees, when Dr. Reese, Chairman of the Committee to whom Dr. Cuthbert's resolutions were referred, made a favorable report. On motion, the report was received and the Committee discharged. These resolutions are of the greatest interest, and are as follows:

WHEREAS, The procuring of criminal abortion is becoming more prevalent, and in our vicinity as elsewhere, unscrupulous specimens of human depravity—abortionists—are plying their murderous traffic; and that from the members of the medical profession the public expect an expression of their opinion in regard to this evil; therefore,

*Resolved*, That we recognize the procuring of miscarriage or abortion in any stage of pregnancy as criminal in the highest degree, unless absolutely demanded to save human life, and then only by the advice of two or more regular physicians.

*Resolved*, That we, as physicians, pledge all our efforts to assist and sustain our legislators and executives in all measures that tend to the suppression and prevention of this heinous crime, with its fearful consequences.

*Resolved*, That we are bound by a sense of duty as physicians and citizens to condemn the abortionist and his abettors, deeming them unworthy of our association and respect, and to use all reasonable means to expose their murderous schemes and bring them to punishment.

*Resolved*, That while we denounce their murderous traffic we will strive to inform the public of its train of consequences; for the violation of physical and moral laws is certain to meet its just penalty, prolonged and intractable diseases of body and mind and often death.

*Resolved*, That a copy of this preamble and resolutions be forwarded to each paper published within our bounds, the Chicago Medical Examiner and *Chicago Medical Journal* inclusive, with a request for gratuitous publication, that the position of the Military Tract Medical Association may be known on this matter of vital importance to the public.

From the standing committees, Practice of Medicine, Surgery, Materia Medica, Obstetrics, Diseases of Women and Children, many interesting and valuable reports were given, both written and oral; the discussions were more free and universal than at any previous meeting.

Under miscellaneous business, Drs. Reese, Judd, Phillips, McClelland, and Webster, were appointed as committee to revise the Constitution and By-Laws. Drs. Phillips, Morse and Hurd were appointed a committee to report at the next meeting upon the death of Dr. John W. Spalding.

The following are the standing committees to report at the next meeting, to be held in January: *Censors*—M. Reese, M. A. McClelland, A. Klingburg. *Essayists*—W. W. L. Cuthbert, J. R. Webster. *Surgery*—M. A. McClelland, J. M. Morse, Wm. Hamilton. *Practice of Medicine*—E. L. Phillips, B. F. Brown, J. C. Secord. *Materia Medica*—Henry B. Upton, D. W. C. Bacon, Chas. Bunce. *Obstetrics and Diseases of Women and Children*—W. H. Heller, D. T. Brown, T. J. Maxwell. *Ophthalmology*—L. S. Lambert.

Upon motion, the association adjourned to meet in Galesburg on the second Tuesday in January next.

HERBERT JUDD, *Sec'y.*

